# Fertility intention of college students responding to the three-child policy in Guangzhou, China: a cross-sectional study

**DOI:** 10.3389/fsoc.2025.1504166

**Published:** 2025-04-02

**Authors:** Xiaona Wang, Chonin Cheang, Xiaoqing Zhong, Shengguang Wu, Sujian Xia

**Affiliations:** ^1^The First Affiliated Hospital, Hainan Medical University, Haikou, China; ^2^Department of Public Health and Preventive Medicine, School of Medicine, Jinan University, Guangzhou, China; ^3^Macau Society for Health Economics, Macau, Macao SAR, China

**Keywords:** fertility intentions, questionnaire, cross-sectional study, college students, China

## Abstract

**Background:**

In recent decades, a noticeable decline in birth rates has been observed globally, particularly in developing countries. Aganist this backdrop, this study investigates fertility intentions and associated factors among college students in Guangzhou, China, within the context of China’s relaxation of the three-child policy in May 2021.

**Methods:**

Between May and July 2021, a cross-sectional survey involving 971 participants was conducted. Participants provided information regarding their demographic characteristics, childbearing preferences, and the factors influencing their fertility plans.

**Results:**

From the data collected, only 43.9% of the participants planned to have children in the future, while 29.8% were unsure, and 26.3% had no intention of having children. It was observed that fertility knowledge among college students in Guangzhou was somewhat limited. Certain factors, like a harmonious family atmosphere, absence of gender preference, and positive peer influences, correlated with higher fertility intentions. However, those who did not perceive fertility as an essential life experience exhibited lower fertility intentions.

**Conclusion:**

Our findings primarily indicate that college students in Guangzhou possess limited fertility knowledge. Although the new fertility policy might be beneficial, there is no guaranteed assurance that it will lead to a rise in fertility rates among this demographic.

## Introduction

Over the past few decades, there has been a marked decline in birth rates across the globe, with an accentuated decrease in developing nations. Fertility is instrumental in shaping population growth, age structure transformations, and lies at the heart of the Program of Action of the International Conference on Population and Development (ICPD). The seventh national census data, released on May 11, 2021, disclosed that China’s elderly population (60 years and above) constituted 18.7% of the total. In 2020, the fertility rate for women of reproductive age in China was a mere 1.3% (China Statistics Press, 2021). This has resulted in both a reduced fertility level and a diminished awareness among the youth regarding pre-conception health and the risks associated with age-driven fertility decline.

Recent times have witnessed an amplified focus on shifts in age patterns of childbearing, overall fertility rates, and marital trends, especially among college-going youth. The fertility dilemmas faced by young college students in China present significant challenges, considering their reproductive phase and the balance they need to strike between family initiation and educational or career pursuits (Chan et al., 2015; Zhou et al., 2020). Due to traditional Chinese values, along with a shortage of educators and appropriate curricula, sexual and fertility health education remains sparse and in its infancy (Dai, 2020; Guo et al., 2019). The two-child policy introduced by China in January 2016 permitted couples to have two offspring. Yet, the anticipated family expansion among couples was not realized, leading to a negligible effect on the nation’s natural population growth rate (Zeng and Hesketh, 2016). In response to the growing concerns of an aging populace, China unveiled a new fertility regulation on May 31, 2021, permitting couples to have up to three children (CPC Central Committee State Council, 2021).

Fertility aspirations can, to a degree, direct fertility actions and mirror an individual’s anticipations concerning their reproductive prospects (Lau et al., 2018; Machiyama et al., 2019). Past research has unveiled a notable concordance between fertility intentions and actual reproductive outcomes (Quesnel-Vallée and Morgan, 2003). Additionally, a recent publication posited that the ramifications of the COVID-19 pandemic on physical and mental wellness might adversely affect people’s fertility plans (Zhu et al., 2020). Thus, interpreting fertility aspirations stands as a reliable metric for predicting broad human reproductive behavior, rendering studies on fertility intentions pivotal (Wang et al., 2019).

International research concerning fertility intentions has predominantly concentrated on the repercussions of women’s societal roles and standing on fertility reduction. European research indicates that socio-economic policies mainly fuel reduced fertility (Hobson and Oláh, 2006; Billari and Kohler, 2004; Yu and Kuo, 2017). In contrast, Chinese inquiries into fertility intentions have largely honed in on the influence of economic assets and pre-conception family backing (Liu et al., 2020; Bao et al., 2014; Wei and Xu, 2020), often segmenting based on occupation, only-child status, non-only child dynamics, or age groupings within the reproductive age bracket.

As per recent data from China’s Ministry of Education, a staggering 35.99 million college students are enrolled in higher education institutions, aged 17 to 27 accounts for approximately 98.70%. This data not only reflects the current situation of higher education in China, but also representing a significant segment of the potential reproductive populace. Analyzing the fertility intentions of college students is, therefore, not only reflective of the broader population’s childbearing aspirations but also offers a window into potential future demographic trajectories. The demographic weight of understanding college students’ reproductive desires is substantial (Delbaere et al., 2021). Nonetheless, scholarly explorations into college students’ fertility inclinations have been scant, leaving a void concerning their fertility aspirations in the wake of the new family planning policy (Chan and Cheang, 2023). The potential impact of this new policy on boosting fertility remains ambiguous. Guangzhou, a global hub of political, economic, and societal exchanges and a beacon of influential outreach, boasts the highest college student population in China, totaling 1.30 million. Addressing this research lacuna, our study endeavors to elucidate three facets: the fertility ambitions of college students over the ensuing 5 to 10 years, the determinants shaping their reproductive choices, and potential governmental strategies to foster reproductive aspirations.

## Results

The cohort for this study was composed of 971 respondents with a mean age of 21.08 ± 1.82 years, spanning ages 17 to 27. The gender distribution consisted of 444 males (45.7%) and 527 females (54.3%). A substantial 72.7% hailed from rural locales, and a significant 58.5% had had romantic engagements. The one-child policy’s legacy was evident, with 38.7% being single children. When exploring their future plans, only 427 participants (43.9%) demonstrated an inclination toward parenting. Notably, among those with fertility intentions, 94.6% expressed a desire to have more than two children. Conversely, 29.8% remained ambivalent, while 26.3% declined the prospect of parenthood. Interestingly, future child gender did not sway 72.6% of the respondents. As for the optimal childbearing age, 38.6% of the pro-parenthood group opted for post-30 (Table 1).

**Table 1:** Sociodemographic characteristics and overall preferences for having children of study participants (*n* = 971).

Characteristics	*n*	%
Age of participants (years, Mean ± SD)	21.08 ± 1.82	
Gender		
Male	444	45.7
Female	527	54.3
Habitual residence		
Urban	265	27.3
Rural	706	72.7
Sexual orientation		
Heterosexuality	850	87.5
Homosexuality	27	2.8
Other	94	9.7
Relationship status		
Had ever been in relationship	332	34.2
In relationship	236	24.3
Never	403	41.5
Interpersonal relationship		
Pretty good	514	52.9
Average	441	45.4
Intense	16	1.7
Only child family		
Yes	376	38.7
No	595	61.3
Marital status of parents		
Married	874	90
Divorced	59	10
Family atmosphere		
Harmony	675	69.5
General	260	26.8
Tension	36	3.7
Annual household income		
Low	105	10.8
Medium	503	51.8
High	363	37.4
The number of ideal children		
1	23	5.4
2	284	66.5
≥ 3	120	28.1
The gender preference		
Only girl	86	20.1
Only boy	31	7.3
No special requirements	310	72.6
Age planning for having childbirth (years)		
<30	107	25.1
≥ 30	165	38.6
Not clear	155	36.3

SD, standard deviation.

Analyzing the sociodemographic factors, fertility intentions remained relatively stable across most variables, with the exceptions being age, gender, sexual orientation, relationship status, and family environment, all of which showed significant associations (*p* < 0.05 for all). Furthermore, fertility intentions demonstrated significant disparities across different fertility perspectives and external influences. These encompassed reliance in senescence, lineage continuation, spiritual solace, significant life junctures, vicariously living through progeny, spousal emotional tethering, parental fiscal backing, attitudes towards offspring, and peer fertility inclinations (all registering *p* < 0.001, Table 2).

**Table 2:** Distribution of fertility intention among respondents by sociodemographic variables, different fertility viewpoint, and external conditions (*n* = 971).

Variables	Intention for childbirth (*n*, %)			*p-value*
	No	Unsure	Yes	
Age (years)^a^	20.9 ± 1.7	21.0 ± 1.6	21.4 ± 1.9	0.001^**^
Gender				
Male	88 (19.8)	123 (27.7)	233 (52.5)	< 0.001^**^
Female	167 (31.7)	166 (31.5)	194 (36.8)	
Habitual residence				
Urban	60 (23.53)	77 (26.64)	128 (29.98)	0.065
Rural	195 (76.47)	212 (73.36)	299 (70.02)	
Sexual orientation				
Heterosexuality	195 (22.9)	257 (30.2)	398 (46.8)	< 0.001^**^
Homosexuality	12 (44.4)	9 (33.3)	6 (22.2)	
Other	48 (51.0)	23 (24.5)	23 (24.5)	
Relationship status				
Had ever been in relationship	93 (28.0)	102 (30.7)	137 (41.3)	0.309
In relationship	51 (21.6)	69 (29.2)	116 (49.2)	
Never	111 (27.5)	118 (29.3)	174 (43.2)	
Interpersonal relationship				
Pretty good	118 (23.0)	144 (28.0)	252 (49.0)	0.012^*^
Average	133 (30.2)	141 (32.0)	167 (37.9)	
Intense	4 (25.0)	4 (25.0)	8 (50.0)	
Only child family				
Yes	103 (27.4)	105 (27.9)	168 (44.7)	0.587
No	152 (25.5)	184 (30.9)	259 (43.5)	
Marital status of parents				
Married	221 (25.3)	262 (30.0)	391 (44.7)	0.052
Divorced	34 (35.1)	27 (27.8)	36 (37.1)	
Family atmosphere				
Harmonious	148 (21.9)	195 (28.9)	332 (49.2)	< 0.001^**^
General	93 (35.8)	84 (32.2)	83 (31.9)	
Tension	14 (38.9)	10 (27.8)	12 (33.3)	
Annual household income				
Low	26 (24.8)	35 (33.3)	44 (41.9)	0.079
Medium	148 (29.4)	145 (28.8)	210 (41.7)	
High	81 (22.3)	109 (30.0)	173 (47.7)	
Lean on in old age				
Disagree	174 (32.3)	166 (30.9)	198 (36.8)	< 0.001^**^
Neutral	54 (18.6)	84 (29.0)	152 (52.4)	
Agree	27 (18.9)	39 (27.3)	77 (53.8)	
Carry on family name				
Disagree	189 (31.7)	188 (31.5)	219 (36.7)	< 0.001^**^
Neutral	43 (18.3)	63 (26.8)	129 (54.9)	
Agree	23 (16.4)	38 (27.2)	79 (56.4)	
Spiritual sustenance				
Disagree	164 (36.9)	128 (28.8)	153 (34.3)	< 0.001^**^
Neutral	51 (20.7)	83 (33.6)	113 (45.7)	
Agree	40 (14.3)	78 (28.0)	161 (57.7)	
Life experience				
Disagree	166 (44.0)	106 (28.0)	106 (28.0)	< 0.001^**^
Neutral	42 (16.2)	93 (35.9)	124 (47.9)	
Agree	47 (14.1)	90 (26.9)	197 (60.0)	
Fulfilling unfinished dreams by their children				
Disagree	191 (30.1)	188 (29.6)	256 (40.3)	< 0.001^**^
Neutral	40 (19.4)	57 (27.7)	109 (52.9)	
Agree	24 (18.5)	44 (33.8)	62 (47.7)	
Emotional bond				
Disagree	156 (33.7)	142 (30.7)	165 (35.6)	< 0.001^**^
Neutral	65 (23.1)	77 (27.4)	139 (49.5)	
Agree	34 (15.0)	70 (30.8)	123 (54.2)	
Whether parents give financial support				
Yes	99 (19.9)	162 (32.5)	237 (47.6)	< 0.001^**^
No	77 (41.6)	46 (24.9)	62 (33.5)	
Unsure	79 (27.4)	81 (28.1)	128 (44.4)	
Attitudes towards children				
Favorite	160 (24.1)	204 (30.7)	300 (45.2)	< 0.001^**^
Dislike	48 (41.4)	31 (26.7)	37 (31.9)	
Neutral	47 (24.6)	54 (28.3)	90 (47.1)	
The influence of peers’ fertility intention				
Positive	102 (35.2)	77 (26.6)	111 (38.3)	< 0.001^**^
Negative	97 (22.3)	131 (30.1)	207 (47.6)	
No influence	56 (22.8)	81 (32.9)	109 (44.3)	

*p < 0.05, **p < 0.001; a. variance test.

Upon deeper examination, certain factors distinctly steered individuals towards a heightened inclination for childbearing. These driving forces encompass:

*Being male*: Men in the study showed a higher probability of desiring offspring, as evidenced by the odds ratio (OR) of 1.611 (*p* = 0.002).

*A harmonious family setting*: Individuals who come from cohesive family backgrounds exhibited a stronger proclivity to have children, with an OR of 2.078 (p < 0.001).

*Absence of a specific gender preference*: Participants without a preference for their future child’s gender showed a higher fertility intention, as highlighted by the OR of 0.667 (*p* = 0.005).

*Ambition channeled through offspring*: Those who seek to realize their unfulfilled aspirations through their children also expressed a greater desire to parent, with an OR of 2.248 (*p* = 0.006).

*Desire for a larger family*: An inclination for more than two children amplifies the fertility intention, having an OR of 3.247 (*p* = 0.006).

*Positive peer influence*: Individuals who have peers with positive fertility intentions are also more likely to desire children, as indicated by the OR of 1.564 (*p* = 0.046).

**Figure 1:**
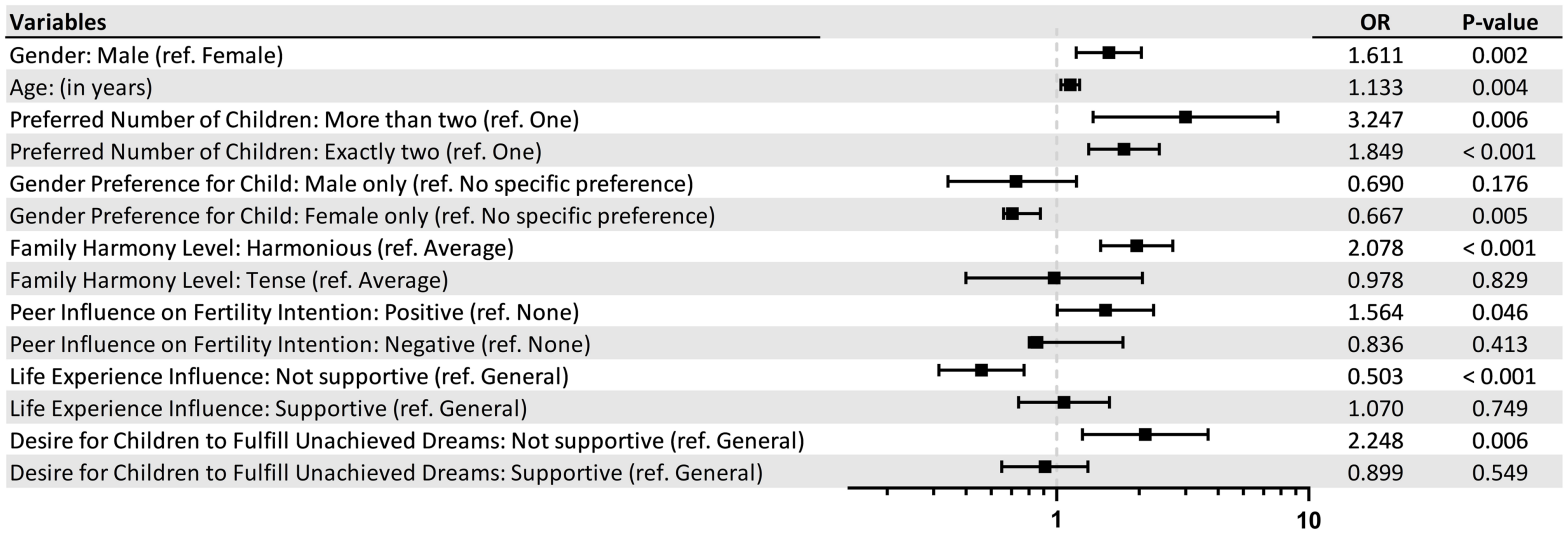
The forest plot of the multivariable logistic regression in the association between childbearing desire and variables.

On the flip side, participants downplaying the significance of parenthood in life’s grand tapestry demonstrated a subdued fertility aspiration. This sentiment is quantified by an OR of 0.503 (*p* < 0.001) for those who do not consider fertility a pivotal life event (as depicted in Figure 1).

## Discussion

The present study explored college students’ fertility intentions under the new fertility policy that allows couples to have three children. It uncovered that only 43.9% of college students were willing to have children. Factors such as gender, age, number of ideal children, gender preferences, family atmosphere, peer influence, and fertility viewpoint were all found to be associated with fertility intentions.

Comparatively, the willingness to give birth in this study is greater than findings from a prior survey in Guizhou, China (Wei and Xu, 2020), yet it is notably lower than the 87.4% recorded in 2014 within Chinese ethnic minority areas (Liang and Wang, 2015). Such variations can be ascribed to methodological discrepancies, geographical differences in the study locations, and the unique childbearing privileges of ethnic minorities. In an international context, 75.0% of Australian college students indicated a preference for childbirth (Delbaere et al., 2021), and the inclination among European college students to have children is similarly robust (Delbaere et al., 2021; Sørensen et al., 2016). The disparity likely stems from regional differences and the timeframe in which these studies were conducted.

This research also highlighted that 94.6% of respondents favored having more than two children, a percentage significantly higher than the 62.3% observed in Shi’s East China study (Shi and Liu, 2018). This preference seems rooted in the experiences of young college students raised during China’s one-child policy era; lacking sibling companionship during their formative years has influenced their reproductive decisions. As a result, many hope to spare their children from a similar solitude and are thus inclined towards larger families (Wang et al., 2019; Zhang et al., 2020).

There exists a gender disparity in fertility intentions, with women exhibiting lower proclivities compared to men. This trend can be attributed to the substantial physical, emotional, and financial burdens that women endure during childbirth and its aftermath (Kaufman, 2000). Such challenges, combined with the potential career interruptions brought about by maternity, can impede women’s professional trajectories (Hashemzadeh et al., 2021). Women pursuing advanced education and personal achievements are particularly susceptible to these deterrents, occasionally resulting in diminished or completely curtailed fertility intentions (Akinyemi and Odimegwu, 2021). To address this, it is imperative to fortify the maternity insurance framework, combat workplace gender biases, and facilitate a conducive environment for women to harmoniously manage both career and family (Hanappi et al., 2017; Chan and Cheang, 2023).

Interestingly, participants without a pronounced gender preference for offspring exhibited heightened fertility intentions, contrasting those with specific gender inclinations. This aligns with studies suggesting that individuals championing gender parity are generally more open to childbearing (Li et al., 2020). In the context of academia, there is a discernible shift among college students, with an eroding bias for male offspring and an emerging appreciation for a diverse gender composition in families (Jiang et al., 2016).

Furthermore, the study accentuates the pivotal roles of familial dynamics and age in shaping fertility perspectives (Ruiyin, 2021; Schaffnit and Sear, 2018). A nurturing family environment fosters affirmative reproductive values in college students, augmenting their receptiveness to parenthood. In stark opposition, a tumultuous household can deter students from the prospect of starting families (Hashemzadeh et al., 2021). Additionally, as individuals age, there is a discernible decline in their fertility inclinations (Hanappi et al., 2017).

Lastly, the influence of peers cannot be understated. Positive peer perspectives on childbearing can significantly bolster the likelihood of college students actualizing their fertility aspirations, a sentiment echoed by multiple studies (Yu and Kuo, 2017; Bernardi and Klärner, 2014; Kuhnt and Trappe, 2016).

## Limitations

This study possesses inherent limitations that warrant mention. To begin, the choice of a commercial street near universities as the survey location may have introduced selection bias, as it predominantly attracted a specific demographic of students. Additionally, the disruptions caused by the novel coronavirus epidemic compromised our data collection process, potentially leading to incomplete participant information. Another constraint is the relatively modest sample size of 971 respondents, which might limit the comprehensiveness of the findings. To enhance the reliability and scope of future findings, it is recommended that multicenter studies with larger sample sizes be employed. While this cross-sectional study furnishes valuable preliminary data on college students’ fertility preferences, it stops short of establishing causative links. Given the evolving nature of fertility intentions over time, continuous monitoring and research are imperative. It would be beneficial to launch longitudinal studies that delve into the interplay of societal, demographic, and attitudinal determinants on fertility inclinations, as well as the alignment between fertility intent and actual outcomes. Notwithstanding these limitations, it is important to note that the study’s sample size satisfies fundamental statistical criteria, capable of discerning a moderately significant effect. As such, it provides a credible reflection of the fertility intentions of college students in Guangzhou.

## Methods

### Study design, setting, and participants

We conducted a cross-sectional study among college students in Guangzhou, China, spanning from May to July 2021. To be considered for the study, participants had to fulfill the following eligibility criteria: (a) Expressed willingness to participate; (b) age over 17 and under 27; (c) no severe physical, mental, or psychological disease; (d) studying in Guangzhou City for more than six months; (e) unmarried; (f) ability to read and write Chinese.

The study secured approval from the Research Ethics Commission at the author’s university. Furthermore, all participants involved in the study furnished their written informed consent prior to participation.

### Sample size

A systematic multi-stage sampling technique was utilized to identify eligible participants. In the initial stage, Guangzhou’s eleven districts were grouped into four regions based on their geographical delineations. Subsequently, a district from each region was randomly picked using a random number generator. In the next phase, prominent commercial streets frequently visited by students were identified in each selected district. These hubs, which include dining, entertainment, and shopping areas, served as our primary sampling units. One commercial street was chosen from each district. Lastly, eligible students from these locales were approached, communicated with, and after mutual consultation, were invited to participate in the study.

To determine the requisite sample size, we employed the formula (Pourhoseingholi et al., 2013) for cross-sectional studies [*N* = z2p (1-p)/e2]. Here, N represents the minimum sample size for a normally distributed population when ‘z’ is 2.68. Drawing from a preceding study (Wang et al., 2019), we presumed a prevalence rate of 54% (*p* = 0.54). The term (1-p) signifies the proportion of individuals with no intention to procreate, and ‘e’ corresponds to a margin of error set at 0.045. Applying this formula yielded a base sample size of 881. However, anticipating a potential 20% non-response, we inflated this figure to 1,057 participants. In the actual study, we managed to obtain a sample size of 971, reflecting a 91.86% response rate. It is pertinent to note that 41 students were excluded as they did not meet the inclusion criteria, and an additional 45 students opted not to complete the questionnaire.

### Procedures

To capture participants’ relevant details, a self-administered questionnaire was crafted, segmented into two primary sections. The inaugural section solicited fundamental sociodemographic details and probed participants’ childbearing inclinations. The succeeding section aimed to discern the influence of fertility intentions, bifurcating into two sub-categories: perspectives on fertility and extraneous influences. Following preliminary testing, necessary modifications were made to enhance the questionnaire’s efficacy. The fertility intention scale’s reliability was gauged via the Cronbach’s alpha coefficient, registering a value of 0.890.

Dependent Variable: Central to our investigation was the fertility intention, quantified by posing, “Do you envisage having children within the forthcoming 5–10 years?” Participants could opt among three potential responses: “No,” “Unsure,” or “Yes.”

Independent Variables: This category encompassed a spectrum of general sociodemographic attributes such as age, gender, habitual domicile, sexual orientation, familial ambiance, current relationship status, family constitution, the marital status of the parents, and the economic status of the household. Aspects of fertility preferences explored encompassed the desired gender of offspring, the envisioned number of progenies, and the prospective age for birthing. Fertility perspectives encompassed considerations like reliance in senescence, the perpetuation of the family lineage, viewing children as a source of spiritual nourishment, children symbolizing pivotal life experiences, the ambition of realizing unfulfilled dreams vicariously through offspring, and the belief that children fortify the emotional bond between spouses. The external determinants scrutinized comprised parental financial backing propensity, their stance on offspring, and the sway of peers’ fertility intentions.

### Statistical analysis

Before analysis, the collected data underwent a meticulous verification process to ensure accuracy and was subsequently entered into the computer system twice for validation. The statistical analyses were facilitated using the SPSS software, version 28.0 (IBM, Armonk, NY, USA). A significance level of *p* < 0.05 was set as the threshold for statistical significance. Descriptive statistics were encapsulated in frequency tables. Continuous metrics were delineated as mean ± standard deviation (SD), while categorical parameters were portrayed in terms of counts (percentages). The chi-square test was employed to discern potential relationships between categorical responses and the array of explanatory variables. To discern the underlying associations between the aspiration for childbearing and various demographic or ancillary variables, multivariable logistic regression was undertaken. For this analysis, fertility intention served as the categorical determinant. Any correlation registering a *p*-value <0.05 was deemed statistically significant.

## Data Availability

The raw data supporting the conclusions of this article will be made available by the authors, without undue reservation.
